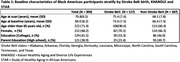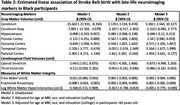# The association of birth in the Stroke Belt and neuroimaging brain biomarkers in a cohort of older Black Americans

**DOI:** 10.1002/alz.090950

**Published:** 2025-01-09

**Authors:** Yi Lor, Kristen M. George, Rachel Peterson, Sirena Gutierrez, Alexander Ivan B. Posis, Charles Decarli, Lisa L. Barnes, Paola Gilsanz, Rachel A. Whitmer

**Affiliations:** ^1^ University of California, Davis, Davis, CA USA; ^2^ University of Montana, Missoula, MT USA; ^3^ University of California San Francisco, San Francisco, CA USA; ^4^ University of California, Davis School of Medicine, Sacramento, CA USA; ^5^ Rush University Medical Center, Chicago, IL USA; ^6^ Kaiser Permanente Northern California Division of Research, Oakland, CA USA

## Abstract

**Background:**

Having ever lived in the southeastern United States, a region referred to as the Stroke Belt (SB), is associated with higher dementia incidence and poorer late‐life cognition. We assessed whether birth in the SB was associated with late‐life MRI‐based regional brain volumes among racially/ethnically diverse older adults who are long‐term residents of Northern California.

**Method:**

KHANDLE and STAR are harmonized cohort studies of long‐term members (ages ≥65 and ≥50, respectively) of an integrated healthcare delivery system. A sub‐sample of participants received MRI imaging, which included regional gray matter and ventricle volumes and total white matter hyperintensities (WMH). The analytic subsample was limited to US‐born Black participants given so few from other ethno‐racial groups were born in the SB. Self‐reported state of birth was dichotomized as birth in the SB or non‐SB. Linear regression models analyzed associations of SB birth with twelve MRI regions of interest, standardized for total cranial volume (except free water and fractional anisotropy). All models adjusted for age at MRI scan, sex, parental education, and own education. Sensitivity analyses were performed in those aged ≥65.

**Result:**

Of 304 participants (mean age=70.8, SD=8.5), one‐third (n=117) were born in the SB (Table 1). Those born in the SB were older (mean age=75.4±7.6), less likely to be college educated (33% vs. 41.5%), and less likely to have parents with a high school diploma (21.4% vs 38.9%). Birth in the SB was associated with smaller parietal cortex volume (β=‐1.82; 95% CI=‐3.31, ‐0.33) (Table 2; Model 2), which remained significant for those who were ≥65 years old (Model 3). Birth in the SB was associated with lower log‐transformed WMH (β=‐0.41; 95% CI=‐0.78, ‐0.03) among those aged ≥65 (Model 3). The associations of other MRI‐derived brain volumes and cognition and cognitive change were null.

**Conclusion:**

Black Americans born in the SB had lower parietal cortex volumes, but also lower WMH volumes in late life. Parietal cortical thinning has been linked with cardiovascular risk, which may warrant further investigation into mechanisms of the association of cardiovascular risk and brain health in those born in the SB.